# Longitudinal Comparison of Three T-Cell Assays and Three Antibody Assays Against SARS-CoV-2 Following Homologous mRNA-1273/mRNA-1273/mRNA-1273 and Heterologous ChAdOx1/ChAdOx1/BNT162b2 Vaccination: A Prospective Cohort in Naïve Healthcare Workers

**DOI:** 10.3390/vaccines12121350

**Published:** 2024-11-29

**Authors:** Hyeyoung Lee, Geon Young Ko, Jihyun Lee, Hyunjoo Bae, Ji Hyeong Ryu, Jin Jung, Hyunhye Kang, Raeseok Lee, Dong-Gun Lee, Eun-Jee Oh

**Affiliations:** 1 Department of Laboratory Medicine, International St. Mary’s Hospital, College of Medicine, Catholic Kwandong University, Incheon 22711, Republic of Korea; shomermaid@catholic.ac.kr; 2 Department of Medical Sciences, Graduate School of The Catholic University of Korea, Seoul 06591, Republic of Korea; geonyoung0107@gmail.com (G.Y.K.); onion1002@naver.com (J.L.); jaydcom8673@gmail.com (H.B.); 3 Department of Laboratory Medicine, Seoul St. Mary’s Hospital, College of Medicine, The Catholic University of Korea, Seoul 06591, Republic of Korea; hyesungsee@naver.com (J.H.R.); jiinj@catholic.ac.kr (J.J.); azuresky@hanmail.net (H.K.); 4 Research and Development Institute for In Vitro Diagnostic Medical Devices, College of Medicine, The Catholic University of Korea, Seoul 06591, Republic of Korea; 5 Division of Infectious Diseases, Department of Internal Medicine, Seoul St. Mary’s Hospital, College of Medicine, The Catholic University of Korea, Seoul 06591, Republic of Korea; misozium03@catholic.ac.kr (R.L.); symonlee@catholic.ac.kr (D.-G.L.); 6 Vaccine Bio Research Institute, College of Medicine, The Catholic University of Korea, Seoul 06591, Republic of Korea

**Keywords:** SARS-CoV-2, vaccine, ELISPOT, interferon-gamma release assay, cellular response, humoral response

## Abstract

**Background**: Cellular and humoral immunity are key to the immune response against SARS-CoV-2, but the comparability and correlation across different assays remain underexplored. This study compares three T-cell and three antibody assays in two vaccine groups. **Methods**: This prospective longitudinal cohort study involved 46 naïve healthcare workers: a total of 11 in the homologous mRNA-1273 group (three doses) and 35 in the heterologous ChAd group (two ChAd doses followed by a BNT booster). Blood samples were collected at five time points. Cellular immunity was assessed using ELISPOT and two commercial interferon-gamma release assays: (IGRA)-QuantiFERON SARS-CoV-2 (QF) and Covi-FERON ELISA (CoVF). Humoral immunity was evaluated using total and IgG antibody assays and a surrogate virus neutralization test. **Results**: The mRNA-1273 group exhibited stronger and more consistent responses than the ChAd group. The correlations between ELISPOT and IGRA varied from weak to moderate (ρ = 0.300–0.410), while QF-IGRA and CoVF-IGRA showed stronger correlations (ρ = 0.700–0.737). The ELISPOT assay showed substantial agreement with QF [Ag2]-IGRA (k = 0.697–0.774) and CoVF [O-sp]-IGRA (k = 0.641–0.718), and an 80.4% agreement rate (k = 0.608) was found between the QF [Ag2]- and CoVF [O-sp]-IGRA tests. Three antibody assays demonstrated very strong correlations with each other and substantial to near-perfect agreement with ELISPOT (k = 0.866–0.949), QF [Ag2]-IGRA (k = 0.807–0.831), and CoVF [O-sp]-IGRA (k = 0.753–0.777). **Conclusions**: SARS-CoV-2-specific cellular and antibody responses vary by platform and vaccine type, highlighting the importance of measuring both T-cell and B-cell responses using multiple assays to comprehensively assess immune status.

## 1. Introduction

Coronavirus disease 2019 (COVID-19), caused by severe acute respiratory syndrome coronavirus-2 (SARS-CoV-2), continues to pose a serious global health challenge [1]. Although vaccination is the most effective strategy for managing COVID-19 [2,3], its protective effect diminishes over time, and the emergence of SARS-CoV-2 variants reduces vaccine efficacy, necessitating booster doses after the primary series [2,4,5,6].

Cellular immunity, particularly T-cell-mediated immunity, plays a pivotal role in the immune response by orchestrating an antigen-specific adaptive defense against infection [7]. For COVID-19, cellular immunity exhibits effective viral clearance, reduced disease severity, and greater long-term stability after vaccination, making the assessment of T-cell-mediated immunity to SARS-CoV-2 crucial for predicting protection against severe disease [8,9]. In contrast, humoral immunity wanes rapidly following vaccination, and a lower humoral immune response is associated with an increased occurrence of breakthrough infections [10,11,12]. Thus, evaluating both cellular and humoral immunity is essential for monitoring protection following SARS-CoV-2 infection or vaccination.

To detect cellular immune responses, various assay platforms can be utilized, including ex vivo interferon-gamma (IFN-γ) enzyme-linked immunosorbent spot (ELISPOT), intracellular staining, activation-induced cell marker, T-cell proliferation assays, and whole blood interferon-gamma release assay (IGRA) [13,14,15]. ELISPOT is widely used for quantifying antigen-specific T-cell responses by measuring cytokine secretion at the single-cell level, allowing for highly sensitive tracking of cellular immunity to targeted antigens [16]. IGRAs measure the production of IFN-γ by effector T cells in whole blood stimulated by previously exposed antigens [15]. They have become the most widespread option for evaluating T-cell immunity due to their commercial availability and simple testing procedure [9,17].

However, a gap remains in fully understanding the immune profiles shaped by different vaccination histories and breakthrough infections [2]. The diversity in immune responses among vaccinated individuals is influenced by multiple factors, such as the cumulative antigen exposure from infections or vaccinations, the vaccination strategies employed, and the specific SARS-CoV-2 strain responsible for the infection [2]. Furthermore, the lack of standardization in T-cell-response assays limits their comparability, and high inter- and intra-assay variability complicates the use of T-cell responses as correlates of protection against COVID-19 [13,18]. Similarly, SARS-CoV-2 humoral immune responses show differences in antibody response levels across assays and reagents [19,20]. Therefore, comparative data on methods for measuring SARS-CoV-2 cellular and humoral immunity are essential for advancing vaccine immunity research [21].

In this prospective longitudinal cohort study of infection-naïve healthcare workers (HCWs), we tested three cellular assays, ELISPOT and two IGRAs (QuantiFERON SARS-CoV-2 and Covi-FERON), as well as three antibody assays, two fully automated chemiluminescent immunoassays (Roche and Siemens) and a surrogate virus neutralization test (sVNT). In South Korea, the Pfizer BioNTech BNT162b2 (BNT) and Oxford AstraZeneca ChAdOx1 nCoV-19 (ChAd) vaccines have been the most widely used, followed by a third dose of mRNA vaccines (BNT or Moderna mRNA-1273) [22]. However, in-depth longitudinal studies that compare homologous (mRNA-1273/mRNA-1273/mRNA-1273) and heterologous (ChAd/ChAd/BNT) vaccination regimens in healthy individuals using multiple tests to assess cellular and humoral responses are limited. This study performed serial evaluations of immune responses for up to three months following the third dose, with the goal of investigating and comparing the serial SARS-CoV-2-specific immune responses in healthcare workers using various assay formats.

## 2. Materials and Methods

### 2.1. Study Design

This prospective longitudinal cohort study included 56 HCWs at Seoul St. Mary’s Hospital to evaluate immune responses following vaccinations. Eligible participants were adult HCWs aged 20 years or older who had never experienced a COVID-19 infection, either currently or in the past. From this cohort, 46 infection-naïve HCWs were selected due to the exclusion of 10 subjects who were lost to follow-up, all of whom had received a two-dose primary vaccination series, followed by a booster between June 2021 and March 2022. No breakthrough infections were reported during the observation period, which lasted for up to three months post-booster. COVID-19 infection status was assessed using self-reported data, including SARS-CoV-2 test results from self-tests or those conducted at other clinics and hospitals, with laboratory confirmation provided when available. Among the participants, 11 received two doses of mRNA-1273, followed by an mRNA-1273 booster (hereafter termed the homologous mRNA-1273 group), and 35 received two doses of the ChAd vaccine followed by a BNT booster (hereafter termed the heterologous ChAd group). Blood samples were collected at five predefined intervals: one month after the second dose (T1), two months after the second dose (T2), three months after the second dose (T3), one month after the booster (T4), and three months after the booster (T5). Table 1 presents the characteristics of the three T-cell assays and three antibody assays used in this study. Trained operators performed the analyses following established protocols, and assay results were verified to ensure consistency and reliability. The Institutional Review Board of Seoul St. Mary’s Hospital approved this study (KC21DIST0174), and informed consent was obtained from all participants.

### 2.2. SARS-CoV-2-Specific Interferon-γ Enzyme-Linked Immunospot Assay

Three overlapping peptide pools covering the surface S protein were used as antigens: PepTivator^®^ SARS-CoV-2 Prot_S [ref 130–126–701] (S), PepTivator^®^ SARS-CoV-2 Prot_S1 [ref 130–127–048] (S1), and PepTivator^®^ SARS-CoV-2 Prot_S+ [ref 130–127–312] (S+) (Miltenyi Biotec, Bergisch Gladbach, Germany). The S peptide pools cover the immunodominant sequence domains at aa 304–338, 421–475, 492–519, 683–707, 741–770, 785–802, and 885–1273. The S+ peptide contains the sequence domain aa 689–895 of the S proteins, while the S1 peptide includes the N-terminal S1 domain (aa sequence 1–692). Patient peripheral blood mononuclear cells (PBMCs) were incubated with these peptides overnight, and an ELISPOT assay was performed as previously described [2,10]. Duplicate or triplicate analyses using frozen PBMCs were conducted after cell viability was confirmed (>80%). All assays were performed according to the validated protocol, and significant outliers, identified by evaluating both negative and positive controls together, were excluded to prevent result distortion. Spots were quantified using an automated AID ImmunoSpot reader and reported as spot-forming units (SFU) per 10^6^ PBMCs, with a test threshold set at 10 SFU/10^6^ PBMCs for each antigen.

### 2.3. SARS-CoV-2-Specific Interferon-γ Release Assay

Two commercial IGRAs were used to detect SARS-CoV-2-specific T-cell responses: QuantiFERON SARS-CoV-2 (Qiagen, Hilden, Germany) and Covi-FERON ELISA (SD Biosensor, Suwon, South Korea). The QuantiFERON SARS-CoV-2 assay (hereafter referred to as QF-IGRA) is an IFN-γ release assay, based on the in vitro stimulation of CD4+ and CD8+ lymphocytes with specific SARS-CoV-2 antigens that cover S protein in heparinized whole blood [23]. IFN-γ production is then measured using ELISA. The assay uses two tubes: SARS-CoV-2 Ag1 (Ag1 tube) and SARS-CoV-2 Ag2 (Ag2 tube). The Ag1 tube contains CD4+ epitopes derived from the S1 subunit of S protein, while the Ag2 tube contains CD4+ and CD8+ epitopes from both the S1 and S2 subunits. For the assay, 1 mL of whole blood was added to each tube and incubated at 37 °C or 16–24 h, and plasma was harvested after centrifuging at 2300× *g* for 15 min. IFN-γ levels were measured using the DS2 ELISA processing system (DYNEX Technologies, Chantilly, VA, USA), with a threshold of 0.15 IU/mL used to classify the samples as positive [18].

The Covi-FERON assay, employing the Covi-FERON ELISA (SD Biosensor, Suwon, South Korea) (hereafter referred to as CoVF-IGRA), detects interferon-gamma (INF-γ) secretion by T cells in response to the SARS-CoV-2 antigen in whole blood samples [24]. This assay includes several components: a Nil tube, a tube with the original spike protein antigen (O-sp), a tube with the variant spike protein antigen (V-sp), a nucleocapsid protein tube for the negative control, and a mitogen tube serving as the positive control. The O-sp tube contains antigens from the original SARS-CoV-2 strain and the B.1.1.7 (20I/501Y.V1) variant, while the V-sp tube contains antigens from the B.1.351 (20H/501.V2) and P.1 (20J/501Y.V3) variants [25]. A total of 1 mL of whole blood was incubated at 37 °C for 16–24 h, and plasma was collected after centrifugation at 2300× *g* for 15 min. IFN-γ levels were measured using ELISA, and the final INF-γ concentrations were calculated by subtracting the Nil tube values [25]. A threshold of 0.25 IU/mL was used to determine the results [26]. All assays were performed in accordance with the manufacturer’s instructions, and ELISAs for IFN-r measurement were performed using harvested plasma in batch.

### 2.4. SARS-CoV-2 Antibody Assays

Antibody levels were assessed using two fully automated chemiluminescent immunoassays: the Elecsys Anti-SARS-CoV-2 assay (Roche Diagnostics, Basel, Switzerland) for total antibodies and the Atellica IM SARS-CoV-2 IgG assay (Siemens Healthineers, Munich, Germany) for IgG antibodies. If antibody levels exceeded the assay limits, the samples were retested after dilution. Roche classified the results as positive for anti-SARS-CoV-2-S antibodies if the levels were 0.8 U/mL or higher, while Siemens considered anti-SARS-CoV-2 IgG antibodies positive at levels of 1.0 U/mL or higher. Binding antibody units per milliliter (BAU/mL) was calculated using conversion factors traceable to WHO international standards for anti-SARS-CoV-2 immunoglobulin (Roche: 1.028; Siemens: 21.803) [20]. Neutralizing antibody (NA) activity against the receptor-binding domain (RBD) of the S protein was determined using the SARS-CoV-2 surrogate virus neutralization test (GenScript, Piscataway, NJ, USA) [27]. A positive threshold of 30% was applied based on the manufacturer’s recommendations. All assays were performed according to the respective manufacturer’s protocols.

### 2.5. Statistical Analysis

Data were analyzed and visualized using GraphPad Prism 10.1.0 for Windows (GraphPad, San Diego, CA, USA). Nonparametric quantitative data were analyzed using the Mann–Whitney U test and Kruskal–Wallis test. Categorical data were expressed as counts and percentages and analyzed using either the chi-squared test or Fisher’s exact test. Correlations were evaluated using Spearman’s correlation coefficient (Spearman’s rho, ρ), with correlation strength categorized as very strong (ρ ≥ 0.8), strong (0.6 ≤ ρ < 0.8), moderate (0.4 ≤ ρ < 0.6), weak (0.2 ≤ ρ < 0.4), or weak to no correlation (ρ < 0.2) [18]. Two-sided p-values < 0.05 were considered significant. Assay agreement was evaluated using Cohen’s kappa statistics and categorized as follows: poor (<0.00), slight (0.00–0.20), fair (0.21–0.40), moderate (0.41–0.60), substantial (0.61–0.80), or nearly perfect (0.81–1.00) [28]. The Wilcoxon matched-pairs signature rank test was used to compare results between different peptide stimuli (ELISPOT; S, S1 vs. S+, QF-IGRA; Ag1 vs. Ag2, CoVF-IGRA; O-sp vs. V-sp).

## 3. Results

### 3.1. Study Population

The demographic characteristics of this study’s population are outlined in Table 2. The participants included six males and forty females, with a median age of 36 years (range: 23 to 57 years). The homologous mRNA-1273 group was younger than the heterologous ChAd group (mean age 29.1 vs. 39.2 years, *p* = 0.0004). During the follow-up period, 3–7 months after the third dose, SARS-CoV-2 breakthrough infections were confirmed in 63.7% of the homologous mRNA-1273 group and 42.9% of the heterologous ChAd group. The median interval between the second vaccine dose and the third booster was 161 days in the homologous mRNA-1273 group and 167 days in the heterologous ChAd group. We compared immune responses between the individuals who experienced breakthrough infection and those who did not and found no significant differences in our study.

### 3.2. Longitudinal SARS-CoV-2-Specific ELISPOT Responses in the Two Vaccine Groups

**Figure 1:**
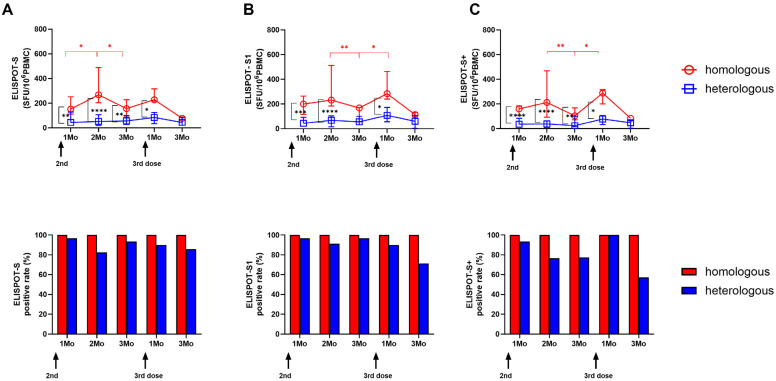
Longitudinal SARS-CoV-2-specific ELISPOT responses against three peptide pools in each vaccine group. SARS-CoV-2-specific ELISPOT responses against S (**A**), S1 (**B**), and S+ (**C**) peptides along with the corresponding positive rates are presented. The Mann–Whitney test was used for the significant changes over the study period. * *p* < 0.05; ** *p* < 0.01; *** *p* < 0.001; **** *p* < 0.0001.

We compared longitudinal cellular responses using SARS-CoV-2-specific ELISPOT assays targeting the S (Figure 1A), S1 (Figure 1B), and S+ peptide pools (Figure 1C) in the two vaccine groups. In the homologous mRNA-1273 group, ELISPOT responses increased up to 2 months after the second dose but significantly decreased by 3 months (0.3-fold to 0.6-fold, *p* < 0.05). After the third booster, a 1.5-fold to 2.8-fold increase in ELISPOT response was observed 1 month post-booster. In contrast, the heterologous ChAd group showed sustained responses throughout the study, with a peak at 1 month following the third BNT booster. When comparing the ELISPOT results between the two groups at each sampling point, the homologous mRNA-1273 group showed higher responses than the heterologous ChAd group until 1 month after the third dose (*p* < 0.05). We also compared ELISPOT positive rates using a cut-off defined as >10 SFU/10^6^ PBMCs. All participants in the homologous mRNA-1273 group showed positive ELISPOT responses throughout the study. In contrast, the heterologous ChAd group showed a decline in positivity, ranging from 76.5 to 96.8%, following the second dose and decreasing to 57.1–85.7% at 3 months post-third dose.

### 3.3. Longitudinal SARS-CoV-2-Specific Interferon-Gamma Release Assays in the Two Vaccine Groups

**Figure 2:**
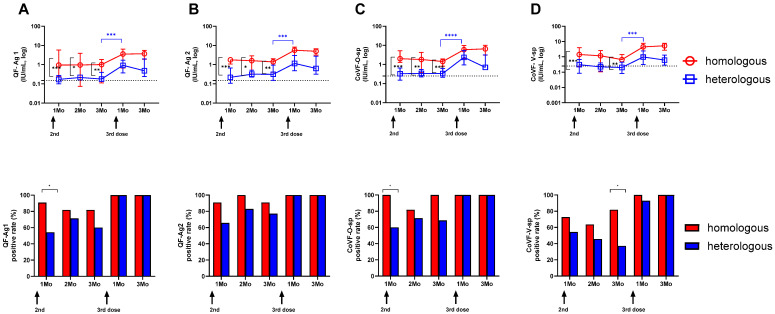
Longitudinal changes in SARS-CoV-2-specific interferon-gamma release assays. SARS- CoV-2-specific cellular responses were measured with the Ag1 (**A**) and Ag2 (**B**) tubes in QuantiFERON and the original spike (**C**) and variant spike (**D**) tubes in Covi-FERON. The Mann–Whitney test was used for the significant changes over the study period. Positive rates were compared using the chi-square or Fisher’s exact test. * *p* < 0.05; ** *p* < 0.01; *** *p* < 0.001; **** *p* < 0.0001.

Longitudinal cellular responses using SARS-CoV-2-specific IGRA assays between the two vaccine groups are shown in Figure 2. We compared the IGRA response measured with the Ag1 (Figure 2A) and Ag2 (Figure 2B) tubes in QuantiFERON and the original spike (Figure 2C) and variant spike (Figure 2D) tubes in Covi-FERON. In both groups, the IGRA responses remained sustained throughout the study, increasing after the third doses. The homologous mRNA-1273 group consistently showed higher IGRA levels than the heterologous ChAd group up to 3 months after the second dose (*p* < 0.05). The IGRA positivity rates were also compared using cut-offs of 0.15 IU/mL for QF-IGRA and 0.25 IU/mL for CoVF-IGRA. After the second dose, the mRNA-1273 group had higher positivity rates until 3 months post-dose, but following the third dose, most participants in both groups exhibited positive IGRA responses.

### 3.4. Longitudinal SARS-CoV-2-Specific Antibody Levels

**Figure 3:**
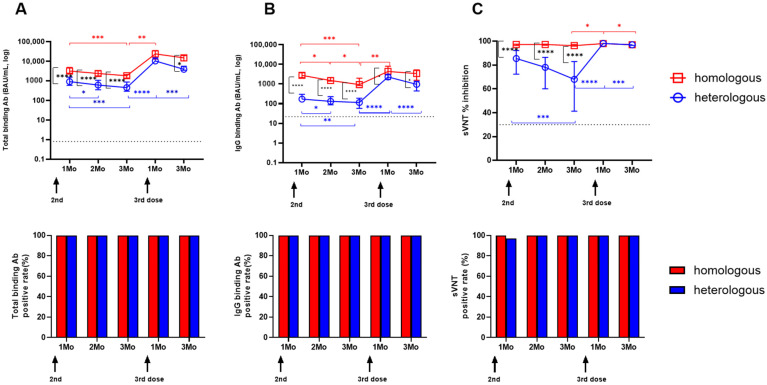
Longitudinal changes in three antibody assays. SARS-CoV-2-spike-specific total binding antibody levels (**A**), IgG antibody levels (**B**), and neutralizing antibody levels (**C**) from the SARS-CoV-2 surrogate virus neutralization test are presented. The assay cut-off is shown as a horizontal dashed line. The Mann–Whitney test was used for the significant changes over the study period. * *p* < 0.05; ** *p* < 0.01; *** *p* < 0.001; **** *p* < 0.0001.

Both vaccine groups showed a significant drop in total and IgG antibody levels 3 months after the second dose compared to 1 month after the second dose (Figure 3A,B). In the homologous mRNA-1273 group, median total antibodies fell from 3177.5 to 1834.9 (*p* = 0.0004), and IgG levels fell from 2744.9 to 934.7 (*p* = 0.0003). In the heterologous ChAd group, total antibodies dropped from 913.9 to 452.3 (*p* = 0.0009), and IgG levels dropped from 170.9 to 114.7 (*p* = 0.0026). After the third booster, antibody levels significantly increased in both groups (*p* < 0.0001). Throughout the study, the homologous mRNA-1273 group showed higher antibody levels than the heterologous ChAd group in both assays (*p* < 0.05) except for 1 month after the third booster dose. Regarding NA activities measured by sVNT, the homologous mRNA-1273 group maintained consistently high levels (>95%) throughout the study period (Figure 3C). In contrast, the heterologous ChAd group showed a significant decline in NA activity from 1 month to 3 months after the second dose (85.5% [48.2–97.7] vs. 68.0% [25.6–97.1], *p* = 0.002). After the third dose, both groups showed a significant increase in NA activity compared to 3 months post-second dose (*p* < 0.05). When we analyzed the positive rates of binding antibody levels, both groups showed 100% positivity until 3 months post-third dose. NA activities were also positive throughout the study period except for 1 month after the second dose in the heterologous ChAd group (97.1%).

### 3.5. Correlation and Agreement Between Three SARS-CoV-2-Specific Cellular Assays (ELISPOT, QF-IGRA, CoVF-IGRA)

**Figure 4:**
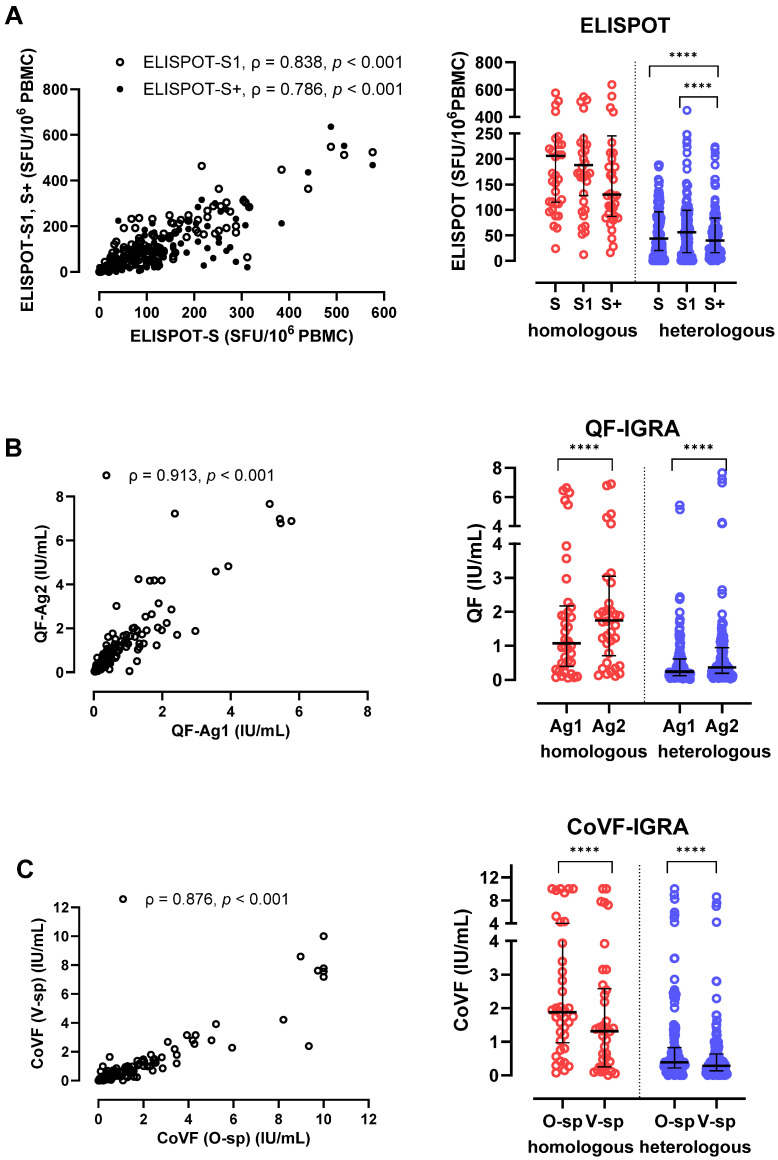
Correlations between SARS-CoV-2 T-cell assays using the same platforms but different peptide stimulations. Correlation between three peptides of ELISPOT (**A**), correlation between Ag1 tube and Ag2 tube in QF-IGRA (**B**), and correlation between O-sp tube and V-sp tube in CoVF-IGRA (**C**) are presented. **** *p* < 0.0001.

The three cellular tests, using the same platforms but different peptide stimulations, demonstrated strong correlations (Figure 4). S, S1, and S+ ELISPOT results showed strong correlations (ρ = 0.786–0.838), with higher ELISPOT results for S1 > S+ > S in the heterologous ChAd groups (*p* < 0.001) (Figure 4A). Similarly, QF-IGRA showed a strong correlation between the Ag1 and Ag2 stimulations (ρ = 0.913), with both vaccine groups showing higher Ag2 tests (*p* < 0.001) (Figure 4B). CoVF-IGRA also showed a strong correlation between O-sp and V-sp (ρ = 0.876), with O-sp inducing higher responses (*p* < 0.001) (Figure 4C).

**Figure 5:**
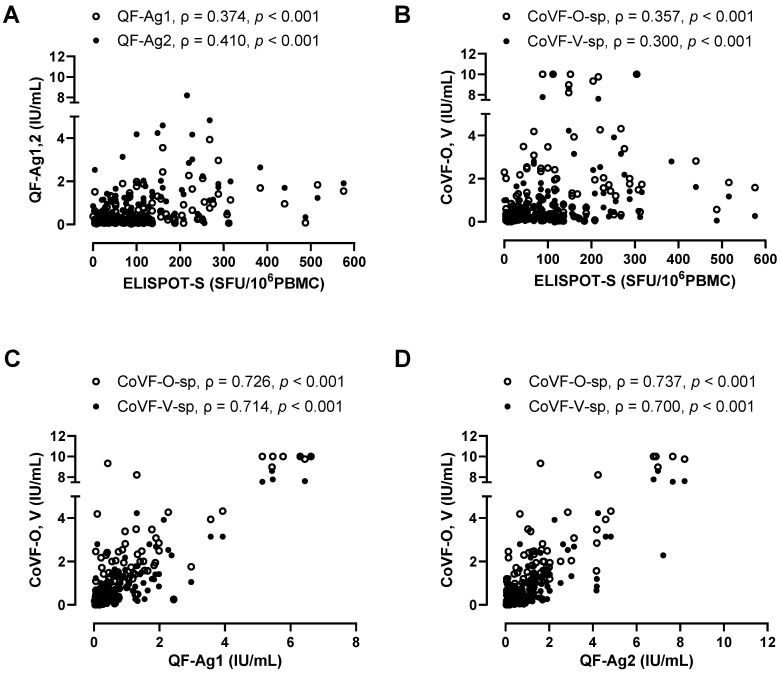
Correlation between SARS-CoV-2-specific ELISPOT and IGRAs. Correlation between ELISPOT and QF-IGRA (**A**), correlation between ELISPOT and CoVF-IGRA (**B**), correlation between Ag1 tube in QF-IGRA and CoVF-IGRA (**C**), and correlation between Ag2 tube in QF-IGRA and CoVF-IGRA (**D**) are presented.

When correlating ELISPOT results with QF- or CoVF-IGRA results, weak to moderate correlations were observed (ρ = 0.300–0.410) (Figure 5A,B). QF [Ag1]-IGRA and QF [Ag2]-IGRA showed strong correlations with CoVF-IGRA (Figure 5C,D).

**Figure 6:**
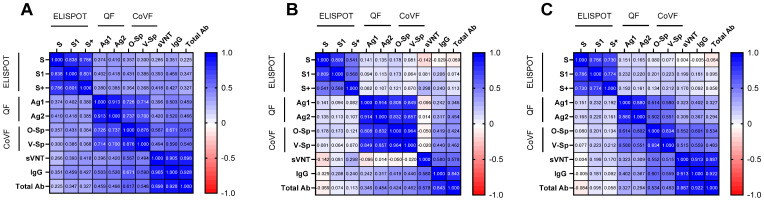
Correlogram of SARS-CoV-2-specific cellular and humoral response measured by ELISPOT, IGRA, and antibody assay and neutralizing antibody assay in total (**A**), homologous mRNA group (**B**), and heterologous ChAd group (**C**). The intensity of the color represents Spearman’s rank correlation coefficient.

The correlogram between SARS-CoV-2-specific ELISPOT against three peptides, two IGRAs against each of two different stimulants, and two binding antibody assays and the NA assay is shown in Figure 6. QF-IGRA and CoVF-IGRA showed strong correlations (ρ = 0.700–0.737) in total participants (Figure 6A). And a higher correlation in the homologous mRNA group (ρ = 0.808–0.857) (Figure 6B) was observed compared to the heterologous ChAd group (ρ = 0.551–0.614) (Figure 6C).

Agreement rates between peptide stimulations were a range from 89.7% to 93.7% for ELISPOT, 78.0% for QF-IGRA, and 68.7% for CoVF-IGRA (Table 3). The ELISPOT assay showed substantial agreement with QF [Ag2]-IGRA (k = 0.697–0.774) and CoVF [O-sp]-IGRA (k = 0.641–0.718), with higher agreement in the homologous mRNA group than the heterologous ChAd group (Supplemental Table S1). Ag2 stimulation in QF-IGRA and O-sp stimulation in CoVF-IGRA showed higher agreement with ELISPOT compared to Ag1 and V-sp stimulations, respectively. An agreement rate of 80.4% (k = 0.608) was found between the QF [Ag2]- and CoVF [O-sp]-IGRA tests.

### 3.6. Correlation and Agreement Between SARS-CoV-2-Specific Antibody Assays and Cellular Assays

**Figure 7:**
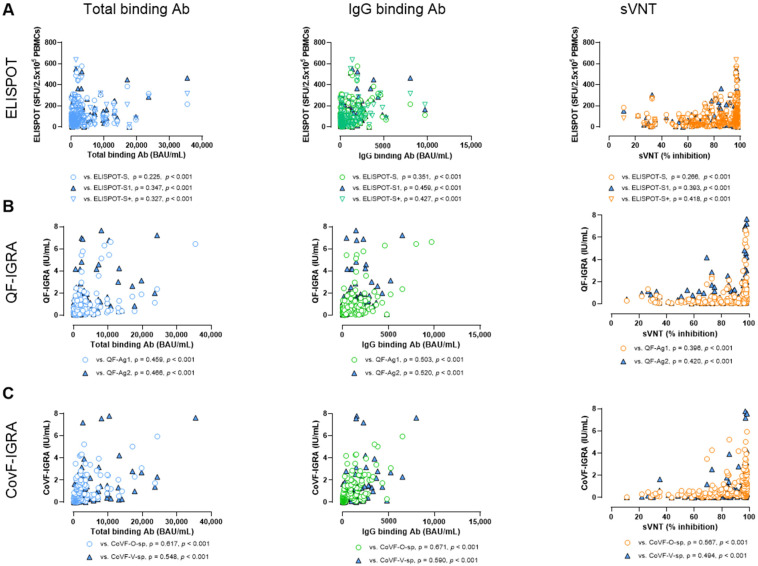
Correlation between SARS-CoV-2-specific antibody assays and three cellular assays. (**A**) Correlation between the three antibody assays and ELISPOT assay. (**B**) Correlation between the three assays and QF-IGRA assay. (**C**) Correlation between the three assays and CoVF-IGRA assay.

The three antibody assays demonstrated very strong correlations with each other (ρ = 0.898–0.928) in total participants (Figure 6A). When comparing the results from the antibody assays and ELISPOT tests, the correlations were weak to moderate (ρ = 0.225–0.459) (Figure 7A). Additionally, when comparing the antibody assays with the two IGRAs, the antibody tests are more closely aligned with IGRA than the ELISPOT assays, showing ρ = 0.396–0.520 for QF-IGRAs (Figure 7B) and ρ = 0.494–0.671 for CoVF-IGRAs (Figure 7C).

The agreement rates among the antibody tests were 98.5–99.7% (Table 3). The antibody assays exhibited nearly perfect agreement with the ELISPOT assays (k = 0.873–0.949 for total antibody assay, k = 0.866–0.924 for IgG assay, and k = 0.848–0.905 for sVNT). Regarding the agreements between the antibody assays and IGRAs, the antibody assays showed nearly perfect agreement with QF [Ag2]-IGRA (k = 0.807–0.831) and substantial agreement with CoVF [O-sp]-IGRAs (k = 0.753–0.777).

## 4. Discussion

Adaptive immunity against viral diseases, including SARS-CoV-2, is driven by both T cells and B cells, offering long-term protection against the virus [29]. During the early stages of the SARS-CoV-2 pandemic, diagnostic efforts focused primarily on testing humoral responses. However, assays for evaluating T-cell-mediated immunity, such as the IFN-γ ELISPOT and IGRA, are now also available [9]. ELISPOT is widely used to assess SARS-CoV-2-specific T-cell responses, using the spike protein as the primary antigen [13]. In contrast, IGRA assesses T-cell immunogenicity by measuring IFN-γ release after stimulating whole blood with specific proteins or peptide pools [30]. QF-IGRA [31,32,33] and CoVF-IGRA [11,34,35] have been established as effective tools for monitoring immune responses in vaccinated individuals.

The primary goal of our study was to compare different cellular and antibody assays in assessing immune responses to SARS-CoV-2 vaccination. However, since immune responses may differ between vaccine platforms, we compared longitudinal immune responses using multiple tests across two vaccine groups. Our longitudinal comparison with multiple assays reflects real-world conditions, where various vaccines and assay methods coexist.

Our findings demonstrate that the homologous mRNA-1273 group consistently exhibited higher cellular responses, particularly in the ELISPOT assay, which more clearly captured the dynamic changes in immune responses. While both groups showed waning immunity 3 months after the second dose, reactivation occurred after the booster dose. ELISPOT positivity was consistently high in the mRNA-1273 group, whereas the heterologous ChAd group showed sustained but lower cellular responses until one month after the third dose. These findings suggest that, while both vaccination strategies effectively elicit cellular immunity, their response kinetics differ, with ELISPOT rather than IGRA detecting these differences more clearly. These findings support previous reports indicating that ELISPOT may have relatively higher sensitivity compared to IGRAs [13,15,36].

Regarding the antibody assays, the homologous mRNA-1273 group demonstrated higher antibody levels and neutralizing activity throughout the study period compared to the heterologous ChAd group. This suggests that, while both vaccine regimens are effective in boosting humoral immunity, the mRNA platform may offer more sustained antibody levels, which are crucial for preventing breakthrough infections [11]. Several studies have demonstrated that mRNA vaccines induce stronger and more persistent immune responses against SARS-CoV-2 compared to vector vaccines. A study by Barbeau DJ. et al. compared mRNA vaccines (BNT162b2, mRNA-1273) with a vector vaccine (ChAd) and showed that mRNA vaccines, particularly mRNA-1273, induced the most durable humoral immune responses and elicited the strongest initial T-cell responses [37]. Additionally, mRNA vaccines achieved higher neutralizing antibody titers against the Delta variant. Further research comparing four different COVID-19 vaccines consistently found that mRNA vaccines elicited higher antibody responses and neutralizing antibodies against both the original SARS-CoV-2 strain and its variants [38]. When comparing the two mRNA vaccines, a recent study revealed that mRNA-1273 shows a more prolonged humoral response compared to BNT162b2 [39]. These findings suggest that the mRNA vaccine may offer advantages in generating robust and adaptable immune responses against SARS-CoV-2. Factors such as age and gender may have influenced these results, as studies indicate cellular immunity increases in older individuals, while humoral responses decline, with higher immune responses typically observed in women [40]. Our study included more females than males, and the mRNA-1273 group was younger, potentially contributing to the differences observed between the groups.

Strong correlations were observed between the peptides used in the cellular assays (ELISPOT; S vs. S1 vs. S+, QF-IGRA; Ag1 vs. Ag2, CoVF-IGRA; O-sp vs. V-sp). However, the ELISPOT and IGRA assays showed weak to moderate correlations, possibly due to differences in the antigens used and assay methodologies: ELISPOT measures IFN-γ-producing antigen-specific T cells, specifically CD4+ and CD8+ T cells, whereas QF-IGRA assesses total IFN-γ production, which may vary across T-cell subsets and individuals depending on factors like lymphocyte counts and immunosuppression [30,36,41].

In the comparison between QF- and CoVF-IGRAs, strong correlations (ρ > 0.7) were found, which contrasts with previous reports reporting a weak to moderate agreement [35]. This discrepancy might be due to differences in the vaccination groups, cut-off levels, or sampling periods. In the homogenous mRNA group, stronger correlations (ρ > 0.8) were observed compared to the heterologous ChAd group (ρ = 0.5–0.6). In addition, QF [Ag2]- and CoVF [O-sp]-IGRAs showed high agreements (80.4%) between different peptide stimulations. The ELISPOT assay showed substantial agreements (k > 0.6) when compared to IGRAs simulated by Ag2 for QF-IGRA and O-sp for CoVF-IGRA. The QF-IGRA Ag1 tube specifically targets S1-specific CD4+ T cells, while the Ag2 tube targets both S1- and S2-specific CD4+ and CD8+ T cells [42]. When we analyzed the IGRA results in pair, both vaccine groups showed higher IGRA results against Ag2 (*p* < 0.0001). This finding aligns with previous studies suggesting that circulating activated memory T cells specific to SARS-CoV-2 may persist longer and elicit a stronger response to Ag2 [43]. Regarding CoVF-IGRA, in line with a previous report [34], the results from the O-sp tube were higher than those from the V-sp tube.

In terms of correlations between the cellular and antibody assays, weak to moderate positive correlations were observed. Previous studies have shown positive correlations between T- and B-cell responses, emphasizing the need for measuring both [44,45,46,47]. Interestingly, the IGRA assays correlated more strongly with antibody responses than ELISPOT. IGRAs, being simpler, more automated, and better suited for large sample volumes, are highly practical for routine diagnostic labs. In the context of waning humoral immunity, T-cell responses measured via IGRA could be a reliable indicator of cellular immunity after SARS-CoV-2 vaccination. However, the lack of standardized cut-off values for IGRA remains a limitation, requiring further validation to establish consistent thresholds that would improve its utility in clinical and research settings [17,48].

T-cell-response kinetics can vary considerably between individuals. The characteristics of study populations contribute to variability in immune responses, and differences in T-cell assessment methods also lead to variation in findings. A recent study has shown that T-cell responses following vaccination can persist longer or peak later than initially expected [49]. Sustained T-cell responses up to 6 months post-vaccination were detected using the ELISPOT assay. This phenomenon may be particularly relevant to the formation of memory T cells [49]. And specific CD4+ and CD8+ T cells remained detectable seven months after a two-dose vaccination, whereas humoral immunity showed signs of impairment as early as 4 months post-vaccination [50].

This study has several limitations. First, we included a small sample size, especially in the homologous mRNA-1273 group, and the follow-up period was limited to only three months post-third dose, which may not fully capture the long-term duration of immunity. Second, COVID-19 breakthrough testing was not systematically performed for all patients, as it primarily relied on self-reported data and/or laboratory confirmation. Consequently, infections with mild symptoms may have been missed. In addition, confounding factors such as age and sex, unmatched groups, as well as the presumed higher exposure risk among healthcare workers may have influenced our results and may limit the generalizability of our findings. We acknowledge that our study design is descriptive in nature and agree that a more conclusive endpoint, such as breakthrough infection or severe disease, could further elucidate the assays’ significance. However, due to the lack of a universally recognized gold standard for these types of COVID-19 assays, our focus was on cross-validation within our sample set. We recognize that establishing a gold standard would allow for a true method comparison, enhancing the interpretation and significance of the assay outcomes. However, the strength of this study is that we provide comprehensive results of the sequential immune response in a homogenous population with the same vaccine strategy. We systematically conducted this study by analyzing samples in the same laboratory on the same platform. This study highlights the distinct dynamics of immune responses elicited by homologous mRNA-1273 and heterologous ChAd vaccine groups, with the mRNA-1273 group exhibiting stronger and more sustained responses in three T-cell assays. Future studies with larger and more diverse populations as well as longer follow-up periods are needed to confirm these findings and better understand the durability of immune responses.

## 5. Conclusions

This comprehensive study comparing immune responses to SARS-CoV-2 vaccination revealed that the homologous mRNA-1273 vaccine group consistently exhibited higher cellular responses and more sustained antibody levels than the heterologous ChAd group. The ELISPOT and IGRA assays showed only weak to moderate correlations, while QF-IGRA and CoVF-IGRA showed stronger correlations. The IGRA assays correlated more strongly with antibody responses than ELISPOT, highlighting the complexity of vaccine-induced immunity. These findings emphasize that SARS-CoV-2-specific immune responses vary by vaccine type and assay method, leading to the importance of using multiple assays for the comprehensive assessment of vaccine responses.

## Figures and Tables

**Table 1:** Characteristics of three SARS-CoV-2 T-cell-response assays and three anti-SARS-CoV-2 antibody assays.

Assay	Platform	Stimulating Agents for T-Cell-Response Assays and Target Epitope for Antibody Assays	Sample	Cut-Off
SARS-CoV-2 ELISPOT assay	Interferon-γ ELISPOT	Peptide pools of S, S1, S+ protein of WT	PBMCs	10 SFU/10^6^ PBMCs
QuantiFERON SARS-CoV-2	Whole blood IGRA	Ag1–CD4+ epitopes derived from the S1 subunit (WT) Ag2–CD4+ and CD8+ epitopes from the S1 and S2 subunits (WT)	Whole blood	0.15 IU/mL
Covi-FERON	Whole blood IGRA	Original spike protein tube—spike protein derived from Wuhan and UK variants (B.1.1.7) Variant spike protein tube—spike protein from South Africa (B.1.351) and Brazil (P.1) strains	Whole blood	0.25 IU/mL
Elecsys Anti-SARS-CoV-2 assay	ECLIA	RBD of S protein	serum	0.8 U/mL
Atellica IM SARS-CoV-2 IgG assay	CLIA	RBD of S1 protein	serum	1.0 U/mL
GenScript SARS-CoV-2 Surrogate Virus Neutralization Test	ELISA	Block the interaction between the RBD with the ACE2 cell surface receptor	serum	30% inhibition

Abbreviations: SARS-CoV-2, severe acute respiratory syndrome coronavirus-2; IGRA, interferon-gamma release assay; ELISPOT, enzyme-linked immunospot; ECLIA, electro-chemiluminescence immunoassay; CLIA, chemiluminescent immunoassay; ELISA, enzyme-linked immunosorbent assay; S, spike; RBD, receptor-binding domain; SFU, spot-forming unit; PBMC, peripheral blood mononuclear cell; WT, wild type.

**Table 2:** Demographic characteristics of this study’s population.

Characteristic	Homologous mRNA-1273 Group (mRNA-1273/mRNA-1273/mRNA-1273) (n = 11)	Heterologous ChAd Group (ChAd/ChAd/BNT) (n = 35)	*p*-Value
Sex, n (%)			0.1124
Female	8 (72.7)	32 (91.4)	
Male	3 (27.3)	3 (8.6)	
Age, years			
mean ± SD	29.1 +/− 6.5	39.2 +/− 7.9	0.0004
group, n (%)			
20–29	6 (63.7)	4 (11.5)	0.0005
30–39	3 (27.3)	16 (45.8)	0.2825
40–49	1 (9.1)	10 (28.8)	0.1874
50–59	0 (0)	5 (14.4)	0.1871
Vaccination schedule			
First to second dose interval (days)			
Median (range)	28 (24–36)	73 (57–91)	
Second to third interval (days)			
Median (range)	161 (154–161)	167 (147–201)	
Breakthrough infection until 7 months after 3rd vaccination, n (%)			
	6 (63.7)	15 (42.9)	0.2335

**Table 3:** Agreement of qualitative results between three T-cell assays and three antibody assays in total participants.

Assay		ELISPOT			QF-IGRA		CoVF-IGRA		sVNT	IgG Antibody
		S	S1	S+	Ag1	Ag2	O-sp	V-sp		
ELISPOT	S1	† 93.7 ‡ (0.874)								
	S+	89.7 (0.795)	90.7 (0.815)							
QF-IGRA	Ag1	82.3 (0.649)	83.3 (0.669)	79.5 (0.592)						
	Ag2	87.7 (0.755)	88.6 (0.774)	84.9 (0.697)	78.0 (0.560)					
CoVF-IGRA	O-sp	84.9 (0.699)	85.8 (0.718)	82.0 (0.641)	75.3 (0.506)	80.4 (0.608)				
	V-sp	75.4 (0.515)	76.3 (0.534)	72.6 (0.458)	66.3 (0.325)	71.4 (0.428)	68.7 (0.373)			
sVNT		95.3 (0.905)	96.2 (0.924)	92.4 (0.848)	85.2 (0.705)	90.4 (0.807)	87.7 (0.753)	78.6 (0.572)		
IgG antibody		96.2 (0.924)	97.2 (0.943)	93.4 (0.866)	86.1 (0.723)	91.3 (0.825)	88.6 (0.771)	79.5 (0.590)	98.5 (0.969)	
Total antibody		96.5 (0.930)	97.5 (0.949)	93.7 (0.873)	86.4 (0.729)	91.6 (0.831)	88.9 (0.777)	79.8 (0.596)	98.8 (0.976)	99.7 (0.994)

Abbreviations: IGRA, interferon-gamma release assay; ELISPOT, enzyme-linked immunospot; QF-IGRA, QuantiFERON SARS-CoV-2 assay; CoVF-IGRA, Covi-FERON ELISA; sVNT, SARS-CoV-2 surrogate virus neutralization test; total antibody, Elecsys Anti-SARS-CoV-2 assay; IgG antibody, Atellica IM SARS-CoV-2 IgG assay. † agreement rate. ‡ Kappa values.

## Data Availability

The data presented in this paper are available on request from the corresponding author.

## Supplementary Materials

The following supporting information can be downloaded at: https://www.mdpi.com/article/10.3390/vaccines12121350/s1, Table S1: Agreement of qualitative results between three SARS-CoV-2 T-cell-response assays and three anti-SARS-CoV-2 antibody assays in homologous mRNA group (A) and heterologous ChAd group (B).
