# Supplementary material for: Longitudinal Comparison of Three T-Cell Assays and Three Antibody Assays Against SARS-CoV-2 Following Homologous mRNA-1273/mRNA-1273/mRNA-1273 and Heterologous ChAdOx1/ChAdOx1/BNT162b2 Vaccination: A Prospective Cohort in Naïve Healthcare Workers
Source: Vaccines (Basel). 2024 Nov 29;12(12):1350. doi: 10.3390/vaccines12121350 (PMC11679843; doi:10.3390/vaccines12121350)
Supplement: Supplementary file 1 [file vaccines-12-01350-s001.zip › vaccines-3291667-supplementary.pdf]

Supplementary Materials

Table S1. Agreement of qualitative results between three SARS-CoV-2 T cell response assays and three anti-SARS-CoV-2 antibody assays

(A) Homologous mRNA-1273 group

Assay		ELISPOT			QF-IGRA		CoVF-IGRA		sVNT	IgG antibody
		S	S1	S+	Ag1	Ag2	O-Sp	V-Sp		
ELISPOT	S1	100† (1.000) ‡								
	S+	100 (1.000)	100 (1.000)							
QF-IGRA	Ag1	93.4 (0.868)	93.4 (0.868)	93.4 (0.868)						
	Ag2	97.4 (0.947)	97.4 (0.947)	97.4 (0.947)	90.8 (0.816)					
CoVF-IGRA	O-sp	97.4 (0.947)	97.4 (0.947)	97.4 (0.947)	90.8 (0.816)	94.7 (0.895)				
	V-sp	88.2 (0.763)	88.2 (0.763)	88.2 (0.763)	81.6 (0.632)	85.5 (0.711)	85.5 (0.711)			
sVNT		100 (1.000)	100 (1.000)	100 (1.000)	93.4 (0.868)	97.4 (0.947)	97.4 (0.947)	88.2 (0.763)		
IgG antibody		100 (1.000)	100 (1.000)	100 (1.000)	93.4 (0.868)	97.4 (0.947)	97.4 (0.947)	88.2 (0.763)	100 (1.000)	
Total antibody		100 (1.000)	100 (1.000)	100 (1.000)	93.4 (0.868)	97.4 (0.947)	97.4 (0.947)	88.2 (0.763)	100 (1.000)	100 (1.000)

(B) Heterologous ChAd group

Assay		ELISPOT			QF-IGRA		CoVF-IGRA		sVNT	IgG antibody
		S	S1	S+	Ag1	Ag2	O-sp	V-sp		
ELISPOT	S1	91.6 (0.832)								
	S+	86.3 (0.726)	87.6 (0.752)							
QF-IGRA	Ag1	78.8 (0.581)	80.1 (0.607)	75.1 (0.505)						
	Ag2	84.6 (0.694)	85.9 (0.719)	80.9 (0.618)	74.2 (0.484)					
CoVF-IGRA	O-Sp	80.9 (0.621)	82.2 (0.647)	77.2 (0.545)	70.7 (0.414)	76.2 (0.523)				
	V-Sp	71.4 (0.439)	72.6 (0.464)	67.6 (0.363)	61.7 (0.234)	67.2 (0.344)	63.7 (0.273)			
sVNT		93.8 (0.875)	95.0 (0.900)	90.0 (0.798)	82.8 (0.656)	88.3 (0.766)	84.8 (0.695)	75.8 (0.516)		
IgG antibody		95.0 (0.900)	96.3 (0.925)	91.3 (0.823	83.9 (0.680)	89.5 (0.789)	85.9 (0.719)	76.9 (0.539)	98.0 (0.961)	
Total antibody		95.4 (0.908)	96.7 (0.933)	91.7 (0.832)	84.4 (0.688)	89.8 (0.797)	86.3 (0.727)	77.3 (0.547)	98.4 (0.969)	99.6 (0.992)

Abbreviations: IGRA, interferon-gamma release assay; ELISPOT, enzyme-linked immunospot; QF-IGRA, QuantiFERON SARS-CoV-2 assay; CoVF-IGRA, Covi-FERON ELISA; sVNT, SARS-CoV-2 surrogate virus neutralization test; total antibody, Elecsys Anti-SARS-CoV-2 assay; IgG antibody, Atellica IM SARS-CoV-2 IgG assay. †agreement rate. ‡Kappa values
